# Supplementary figures and images for: The Gluopsins: Opsins without the Retinal Binding Lysine
Source: Cells. 2022 Aug 6;11(15):2441. doi: 10.3390/cells11152441 (PMC9368030; doi:10.3390/cells11152441)

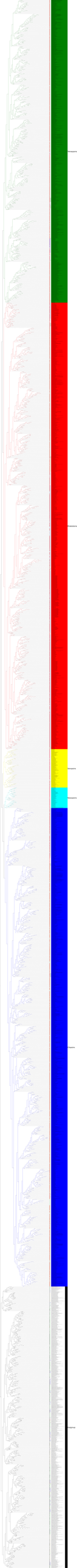

Supplement: Supplementary file 1 [file cells-11-02441-s001.zip › Figure S1 OpsinPhylogeny.pdf]

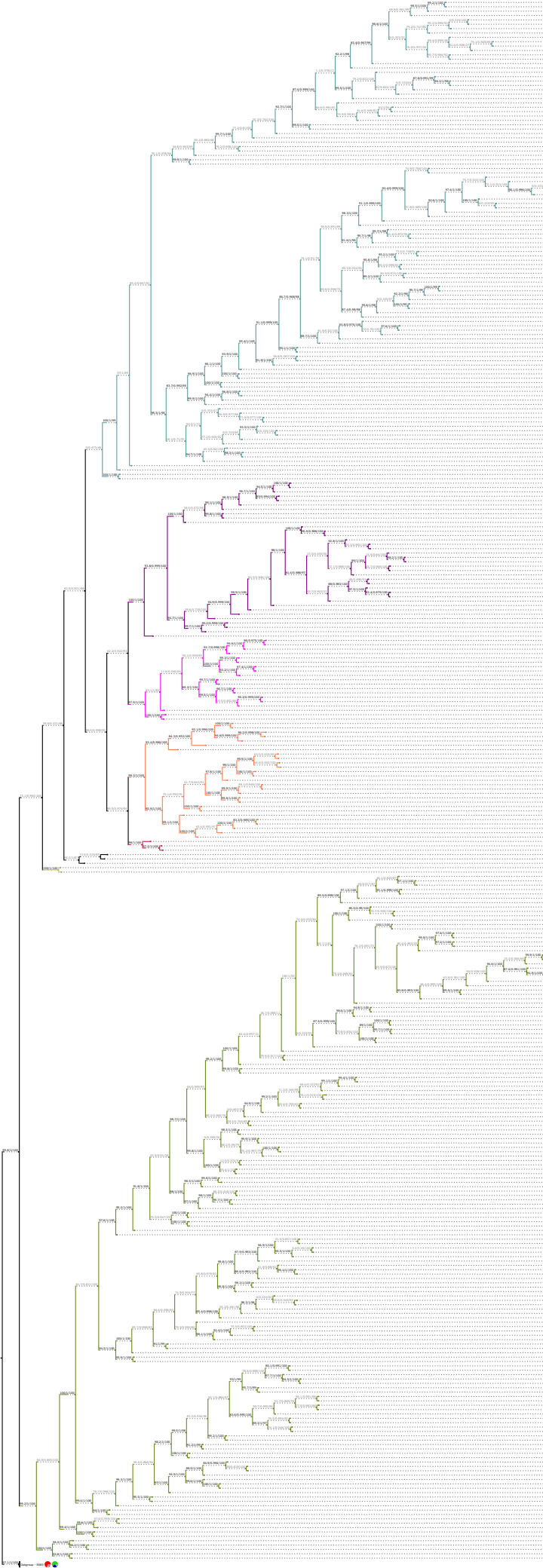

## Peropsins

## Gluopsins

# Varropsins

# Retinochromes

Astropsins  
Nemopsins

## RGR-opsins

Supplement: Supplementary file 1 [file cells-11-02441-s001.zip › Figure S2 ChromopsinPhylogeny.pdf]
